# Influence of wafer quality on chip size-dependent efficiency variation in blue and green micro light-emitting diodes

**DOI:** 10.1038/s41598-022-12169-6

**Published:** 2022-05-13

**Authors:** Kie Young Woo, Hyun Gyu Song, Kwanjae Lee, Young Chul Sim, Yong-Hoon Cho

**Affiliations:** Department of Physics and KI for NanoCentury, Korea Advanced Institute of Science and Technology (KAIST), 291 Daehak-ro, Yuseong-gu, Daejeon, 34141 Republic of Korea

**Keywords:** Materials science, Nanoscience and technology, Optics and photonics

## Abstract

We propose a key factor associated with both surface recombination velocity and radiative efficiency of an LED to estimate its chip size-dependent radiative efficiencies. The validity of the suggested factor is verified through experimental comparison between various LED wafers. Efficiencies of micro-LEDs from a blue and two green LED wafers are examined by temperature-dependent photoluminescence experiments. Surface recombination velocities are extracted from chip size dependent time-resolved PL results. Possible explanations on the reason why two green wafers show different properties are also given. With the suggested factor, we can provide more accurate prediction on the chip size-dependent efficiency of an LED wafer.

## Introduction

Since successful development of InGaN/GaN based blue light emitting diodes (LEDs), LEDs have enlarged their application field and market share extensively, such as indoor/outdoor lighting, signal indicator, interior and exterior art works.^1–4^. For display field, however, the application of the LEDs were restricted to light sources in large panel outdoor display or white backlight sources in LCD-based displays. On the other hand, organic LEDs have long been used as self-emissive display thanks to their good contrast, wide color range, and even flexibility. However, the organic LEDs have limitations to be adopted in emerging portable outdoor display because they are vulnerable to outdoor environment such as heat and humidity^5^. Recently, inorganic semiconductor based micro-LEDs have been attracting much interest because they are applicable to the emerging micro display market with their high efficiency, low power consumption, and tolerance to environmental conditions^5,6^. Especially, there have been notably increasing reports on III-nitride semiconductor based micro-LEDs, since they exhibit high efficiency and brightness, and above all, they can cover all the spectral range that present display requires (e.g. sRGB), in help with color conversion technique^7–9^.

It is reported that reducing lateral dimension of the micro-LEDs accompanies a decrease of their efficiency^10,11^. The main cause of the efficiency decrease has been attributed to be additionally generated non-radiative recombination centers along the perimeter during tough dry etching process^12^, and the degree of the efficiency degradation goes severer for smaller chips as the ratio of perimeter to area increases. These increasing non-radiative centers offer unfavorable additional recombination channel to injected carriers, also known as surface recombination. Moreover, the surface recombination can be also influenced by the properties of active materials such as alloy fluctuation and carrier diffusion^12,13^. Since InGaN-based LEDs with different center wavelength have different material properties in their active region, the degree of efficiency degradation with decreasing chip size would be different from each other even they underwent the same fabrication process.

For example, Kitagwawa et al*.* reported green photonic-crystal LEDs show higher enhancement in light output compared to blue photonic-crystal LEDs, due to smaller surface recombination velocity in green LEDs compared to blue LEDs^14^. The authors suggested enhanced carrier localization in longer wavelength LEDs (i.e. green LEDs) originating from indium segregation inside the quantum wells (QWs) results in smaller surface recombination velocity due to limited diffusivity of carriers. More recently, Smith et al*.* compared size-dependent external quantum efficiency (EQE) for blue and green micro-LEDs with varying diameter down to 1 µm^15^. They suggested that EQE of green micro-LEDs decreases relatively slower compared to that of blue micro-LEDs with reducing the chip size due to smaller surface recombination velocity, eventually at small enough chip size, EQE of green micro-LEDs exceeds that of blue micro-LEDs. However, since the efficiency of an LED is determined by not only the surface recombination velocity but also the radiative and non-radiative recombination of bare LED wafer, the degree of efficiency degradation with reducing chip size could not be determined by the surface recombination velocity only. Moreover, few reports have considered the influence of wafer’s quality on this size dependent efficiency change. Therefore, a detailed investigation on the appropriate factor that determines the size-dependent efficiency of micro-LEDs is required.

In this work, we investigated the influence of wafer-level efficiencies on the chip size-dependent efficiency change in blue and green micro LEDs and suggest a key factor that determines the degree of efficiency degradation with decreasing chip size. For this, the chip size-dependent radiative efficiency of two green LED wafers with different wafer-level efficiencies were compared to that of a conventional blue LED wafer. Time-resolve PL experiments were also performed to extract the surface recombination velocity from chip size-dependent carrier lifetime. The comparison results on the chip size-dependent efficiencies were explained quantitatively by the surface recombination velocity extracted from size-dependent carrier lifetime and the measured recombination rate of the bare LED wafers. The origins that makes two green wafers different are investigated by temperature dependent photoluminescence (PL) measurements and monochromatic cathodoluminescence (CL) images. From these results, an important factor that determines the degree of efficiency degradation with decreasing chip size was found.

## Results and discussion

**Figure 1:**
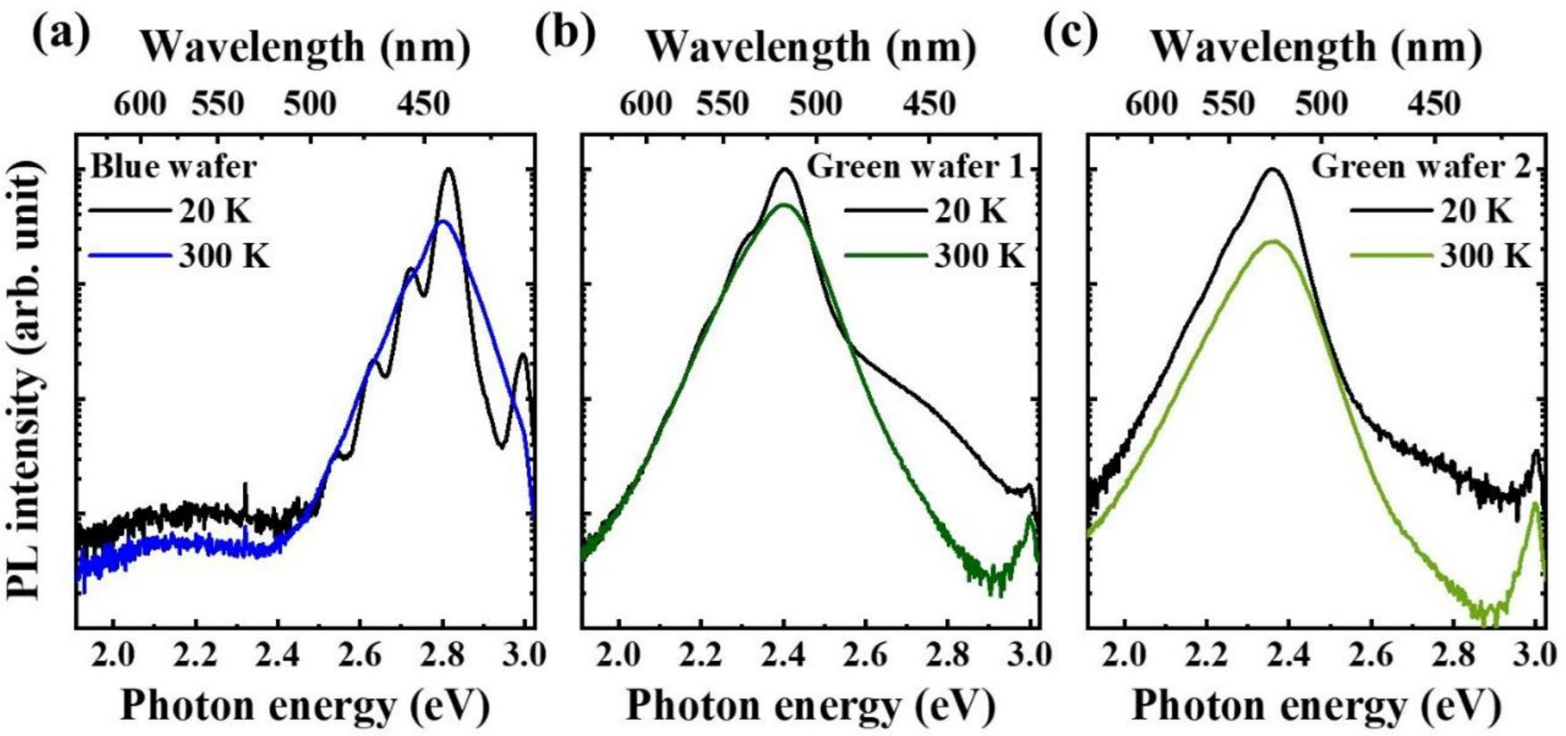
PL spectra taken at 20 K and 300 K for (**a**) blue LED wafer, (**b**) green LED wafer 1, and (**c**) green LED wafer 2.

Figure 1 shows PL spectra of a blue and two green LED wafers taken at 20 K and 300 K. All the samples were excited by a 405-nm pulsed laser with an average power of 200 µW. The blue wafer has center wavelength of 440 nm at 20 K, and 442 nm at 300 K, respectively. At 20 K, photon replica were observed as satellite peaks located at ~ 90 meV intervals from the main peak toward lower energy side. At both 20 and 300 K, the green wafer 1 (2) shows center wavelength of ~ 516 (525) nm. From both green wafers, the phonon replica were less notable than blue wafer due to broader spectra. The PL efficiency at room temperature were then calculated by the ratio of the PL intensity at room temperature to that at 20 K. The calculated PL efficiencies were 75.2% for the blue wafer, and 67.4 and 34.5% for the green wafers 1 and 2, respectively.

**Figure 2:**
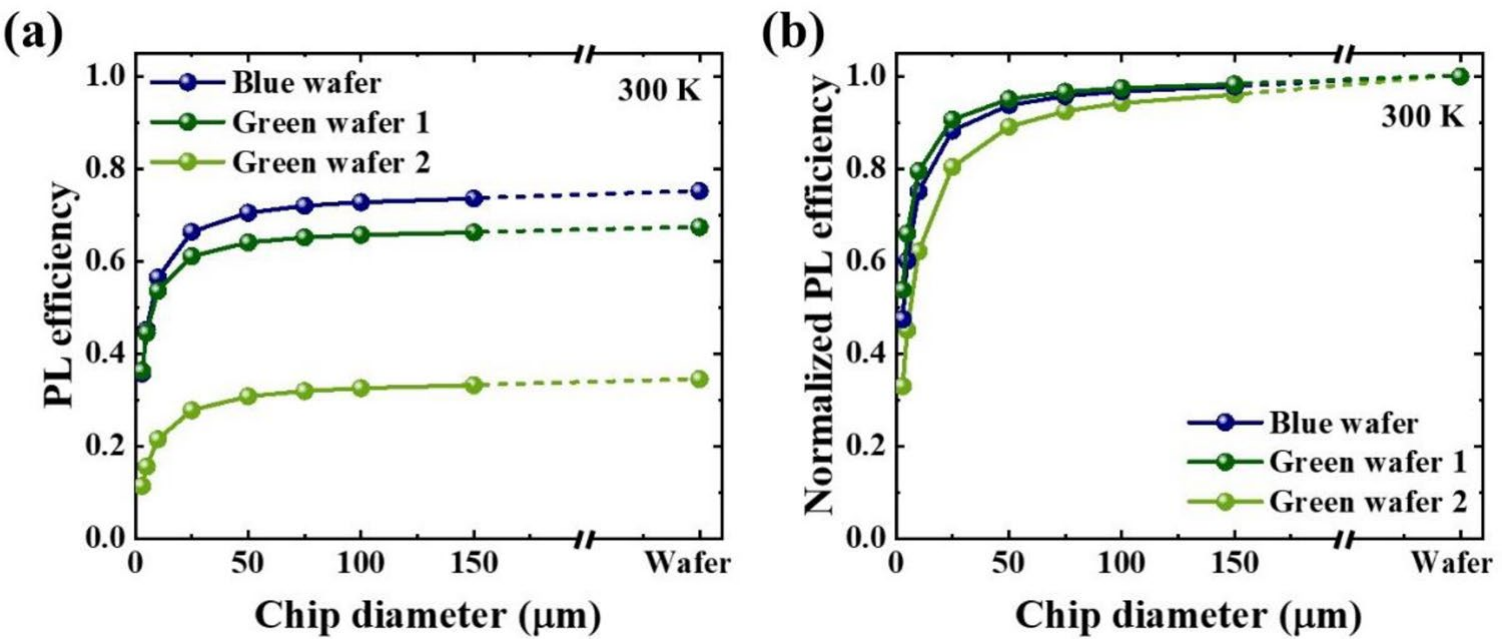
(**a**) Chip diameter dependence of PL efficiencies at room temperature for three examined LED wafers. The PL efficiencies were estimated by the intensity ratio between 20 and 300 K. PL efficiencies of bare wafers are shown together to compare PL efficiencies before and after chip fabrication process. (**b**) Chip diameter dependent PL efficiencies of the three LED wafers normalized by the PL efficiencies of each bare wafer.

PL efficiencies of circular mesa structures with different sizes were also evaluated by the ratio of the PL intensity at room temperature to that at 20 K. Disks with diameter ranging from 3 to 150 µm were fabricated by conventional photolithography followed by dry etching process. For the whole measurements, the optical power of the excitation laser and the spot size were kept constant, so that the optical power density (i.e. the optical power per unit area) was constant. Furthermore, the spot size of the excitation laser was larger than 150 µm and the distance between two adjacent disks are kept as 3 µm, so that at least one disk is pumped by the excitation laser. These conditions make the carrier concentration in various sizes of examined structures constant. PL efficiencies estimated at room temperature for the disks are shown in Fig. 2a. Aforementioned PL efficiencies of bare wafers are shown together for comparison. All three wafers showed a monotonic decrease in PL efficiencies with decreasing chip diameter. When comparing detailed efficiency trend of the blue wafer and the green wafer 1, it could be observed that efficiencies of the blue wafer were higher than those of the green wafer 1 at chip diameter above 5 µm, while the efficiency of the green wafer 1 became higher than the blue wafer at a diameter of 3 µm. However, when comparing between the blue wafer and the green wafer 2, the efficiency of the blue wafer was always higher than that of green wafer 2 at all the diameters we investigated. To investigate the ratio of efficiency reduction with chip diameter, the size-dependent PL efficiencies were normalized to the PL efficiency of each bare wafer, as shown in Fig. 2b. The normalized PL efficiency was always higher for the green wafer 1 than that of the blue wafer, while that of the green wafer 2 was always lower than that of the blue wafer. To verify why this difference between two green wafers happens, further investigation using time-resolved PL experiments is carried out.

**Figure 3:**
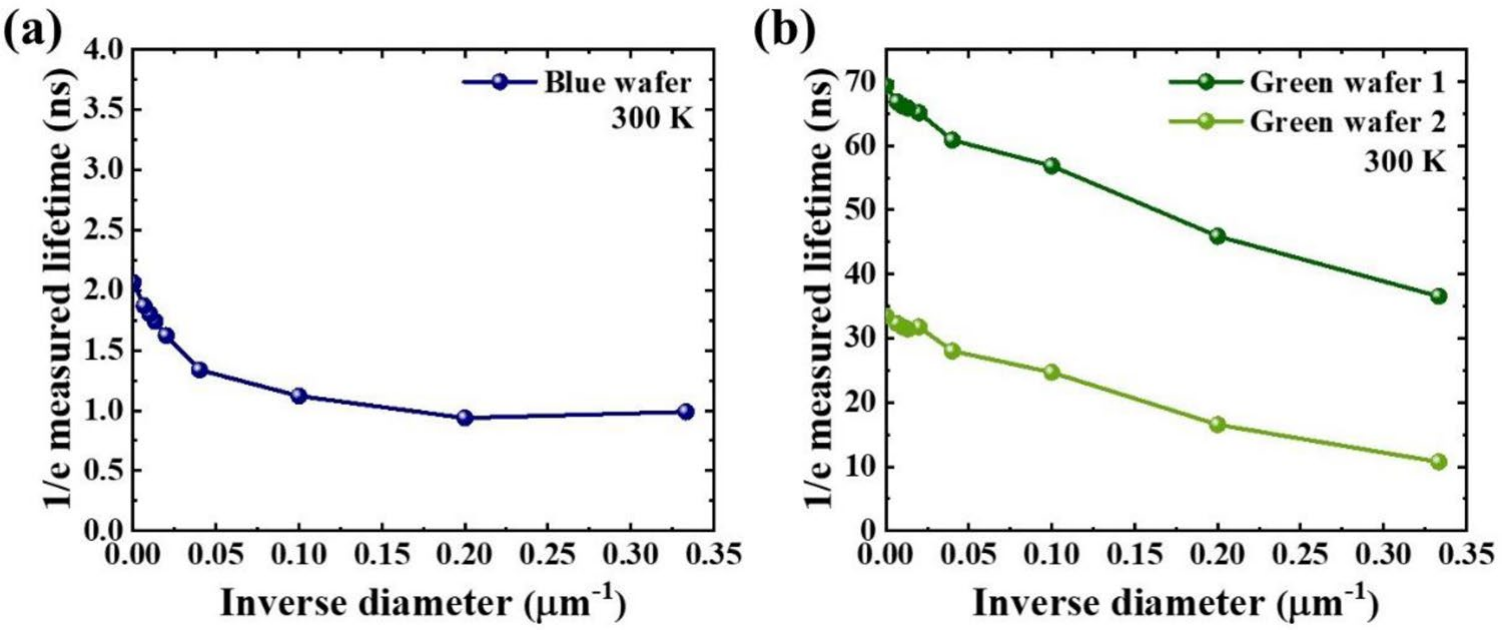
Measured carrier lifetimes as a function of inverse of the chip diameter, which were extracted from the time decay when PL intensity reduces to 1/e of its maximum, for (**a**) blue LED wafer and (**b**) two green LED wafers.

Figure 3 shows chip diameter dependent measured carrier lifetime for three wafers. Measured carrier lifetime for each chip is defined as the time delay when the PL intensity becomes *1/e* of its maximum. For all wafers, the measured lifetime decreases with increasing inverse diameter (i.e. decreasing diameter). This is mainly attributed to increased non-radiative recombination rate with decreasing diameter, which will be discussed later. Surface recombination velocity, ${v}_{s}$, which represents how fast carrier moves toward the perimeter of the disk, can be evaluated by fitting the curve with a formula^14^,

1 $$\tau^{ - 1} \left( D \right) = \tau_{0}^{ - 1} + v_{s} \left( {{{P \, } \mathord{\left/ {\vphantom {{P \, } S}} \right. \kern-\nulldelimiterspace} S}} \right) = \tau_{0}^{ - 1} + v_{s} \left( {{{4 \, } \mathord{\left/ {\vphantom {{4 \, } D}} \right. \kern-\nulldelimiterspace} D}} \right),$$

where ${\tau }^{-1}$ represents reciprocal of the measured lifetime, ${\tau }_{0}^{-1}$ is the reciprocal lifetime at infinite diameter (i.e. bare LED wafer), *P* is length of perimeter, and *S* is top surface area. For disk structure, *P/S* can be rewritten as *4/D*, where *D* represents chip diameter. The resultant surface recombination velocities are 4.02 × 10^4^ cm/s for the blue LED wafer, and 9.29 × 10^2^ and 4.55 × 10^3^ cm/s for the green LED wafers 1 and 2, respectively. It has been reported that surface recombination velocity of nitride materials lie in the range from ~ 3 × 10^2^ to ~ 10^4^ cm/s^14,16^. In addition, it also has been reported that green LED wafers show smaller surface recombination velocity than blue LED wafers^12,14^. In this sense, the surface recombination velocities extracted in this work are in the range of the previously reported values.

As predicted by previously reported papers^12,14^, both the green wafers showed lower surface recombination velocities than the blue LED wafer. However, as shown in Fig. 2, the normalized PL efficiency was always higher for green wafer 1 than that of blue wafer, while that of green wafer 2 was always lower than that of blue wafer. This implies that which wafer would show the higher normalized PL efficiency cannot solely determined by the surface recombination velocity alone. Thus, we tried to find the key factor, which determines the normalized PL efficiency at a certain chip size more precisely, from the formula as follows. Firstly, radiative efficiency for each *D* when the surface recombination presents is expressed as

2 $$\eta = \frac{{\tau_{r}^{ - 1} }}{{\tau^{ - 1} }} = \frac{{\tau_{r}^{ - 1} }}{{\tau_{0}^{ - 1} + v_{s} \left( {{{4 \, } \mathord{\left/ {\vphantom {{4 \, } D}} \right. \kern-\nulldelimiterspace} D}} \right)}},$$

where $\upeta$ represents the radiative efficiency, ${\tau }_{r}^{-1}$ is radiative recombination rate. After normalizing it to efficiency of each bare LED wafer (i.e. $D=\infty$), the normalized efficiency becomes

3 $$\eta_{{{\text{norm}}}} = \frac{{\tau_{0}^{ - 1} }}{{\tau_{0}^{ - 1} + v_{s} \left( {{{4 \, } \mathord{\left/ {\vphantom {{4 \, } D}} \right. \kern-\nulldelimiterspace} D}} \right)}}$$

Taking the reciprocal of both sides,

4 $$\frac{1}{{\eta_{{{\text{norm}}}} }} = \frac{{\tau_{0}^{ - 1} + v_{s} \left( {{{4 \, } \mathord{\left/ {\vphantom {{4 \, } D}} \right. \kern-\nulldelimiterspace} D}} \right)}}{{\tau_{0}^{ - 1} }} = 1 + \frac{{v_{s} }}{{\tau_{0}^{ - 1} }}\left( {{{4 \, } \mathord{\left/ {\vphantom {{4 \, } D}} \right. \kern-\nulldelimiterspace} D}} \right).$$

As a result, normalized efficiency at a certain chip diameter is determined by the factor *v*_*s*_/${\tau }_{0}^{-1}$, and the normalized efficiency becomes higher when this factor is lower. Here, the denominator represents the total recombination rate of a bare wafer, and can be easily calculated by the reciprocal of the measured lifetime of a bare wafer. The measured recombination lifetimes of each bare wafer, surface recombination velocities, and the factor *v*_*s*_/${\tau }_{0}^{-1}$ for three examined wafers are summarized in Table 1.

**Table 1:** Measured carrier lifetime of each bare wafer, surface recombination velocity, and suggested factor, ${\text{v}}_{\text{s}}/{\tau }_{0}^{-1}$, for three examined LED wafers.

	Blue LED wafer	Green LED wafer 1	Green LED wafer 2
${\tau }_{0} \; (\text{ns})$	2.064	69.343	33.484
${\text{v}}_{\text{s}} \;(\text{cm}/\text{s})$	4.02 × 10^4^	9.29 × 10^2^	4.55 × 10^3^
$\frac{{\text{v}}_{\text{s}}}{{\tau }_{0}^{-1}} \;(\text{cm})$	8.30 × 10^–5^	6.44 × 10^–5^	15.2 × 10^–5^

It can be seen that

5 $$\left( {\frac{{v_{s} }}{{\tau_{0}^{ - 1} }}} \right)_{{\text{green wafer 2}}} > \left( {\frac{{v_{s} }}{{\tau_{0}^{ - 1} }}} \right)_{{\text{blue wafer}}} > \left( {\frac{{v_{s} }}{{\tau_{0}^{ - 1} }}} \right)_{{\text{green wafer 1}}} ,$$

which shows an excellent agreement with

6 $$\eta_{{\text{norm, green wafer 2}}} < \eta_{{\text{norm,blue wafer}}} < \eta_{{\text{norm, green wafer 1}}} ,$$

for each chip diameter examined, as shown in Fig.2b.

From these formula, we can conclude that the size dependent efficiencies of micro-LEDs depend on the ratio of surface recombination velocity of chip structure to the reciprocal of measured lifetime of each bare wafer, instead of the surface recombination velocity alone that is previously suggested in literatures.

To understand what makes two green wafers have different surface recombination velocities and total recombination rates, more detail investigation on the bare wafers are carried out. From size-dependent PL efficiency in Fig. 2 and size-dependent measure lifetime in Fig. 3, we could extract size-dependent radiative and non-radiative lifetimes as follows.

$$\tau_{r} = \frac{1}{\eta } \times \tau_{measured} ,$$

7 $$\tau_{nr} = \frac{1}{1 - \eta } \times \tau_{measured} .$$

**Figure 4:**
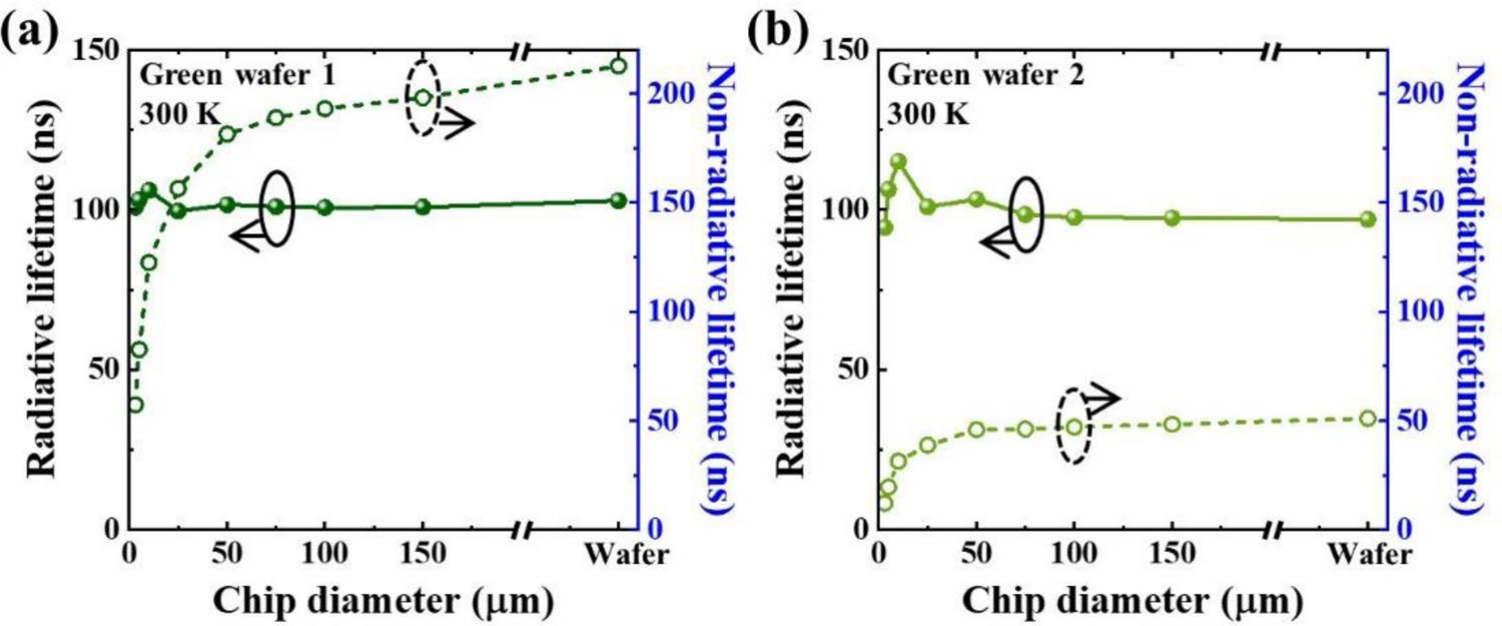
Chip diameter dependent radiative (solid line) and non-radiative (dashed line) carrier lifetimes for (**a**) green LED wafer 1 and (**b**) green LED wafer 2, which were calculated with measured carrier lifetimes and PL efficiencies. Radiative and non-radiative carrier lifetimes for each bare wafer is also shown for comparison.

Figure 4a,b show the size-dependent radiative and non-radiative lifetimes for the green wafers 1 and 2, respectively. Both wafers showed relatively independent radiative lifetime to chip diameter except for a small variation in small size regime. On the other hand, the non-radiative lifetime showed clearly decreasing trends with decreasing chip diameter, for both wafers. These implies that as the chip size decreases, additional non-radiative channels along the perimeter involve, being responsible for the decrease in radiative efficiency. When comparing the green wafers 1 and 2, the radiative lifetime for both wafers are similar to each other. This implies that radiative process does not critically affect the difference between two wafers. However, non-radiative lifetime of the green wafer 2 was much lower than that of the green wafer 1. The origin of the notable difference in non-radiative lifetime is investigated further.

**Figure 5:**
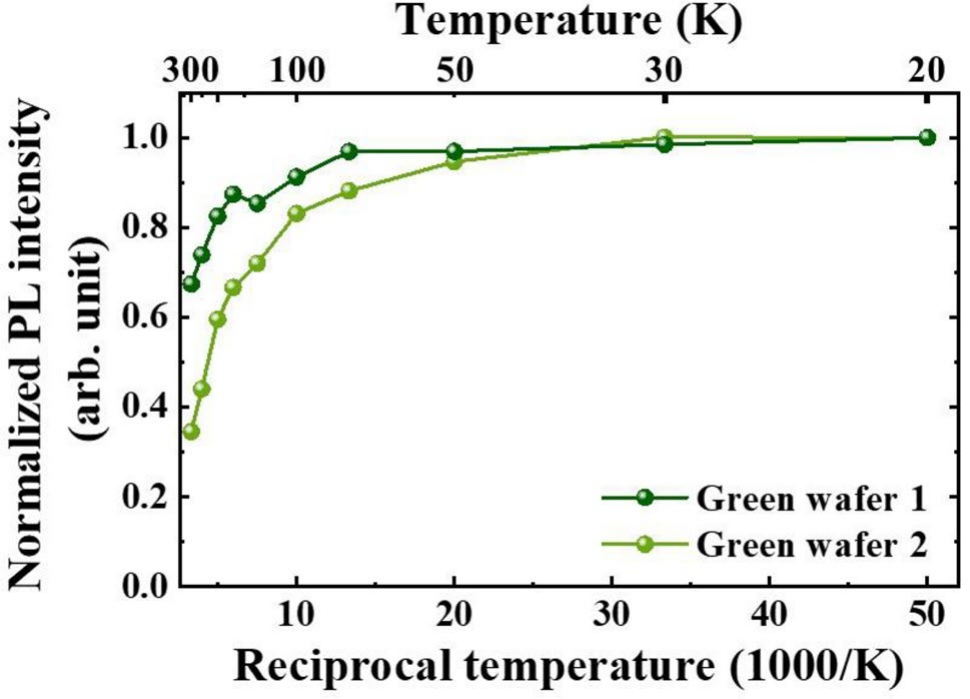
Temperature dependent PL intensity normalized to PL intensity measured at 20 K for green LED wafers 1 and 2.

Temperature dependent PL measurements are carried out to characterize the effective potential barrier for escaping carriers inside QWs. Figure 5 shows temperature dependent PL intensity normalized to the value at 20 K for both green bare wafers. By fitting the curve in T > 150 K region with Arrhenius formula, the thermal activation energy for green wafers 1 and 2 were extracted as 54.01 and 40.96 meV, respectively. Higher activation energy for green wafer 1 implies stronger confinement in localized centers and less accessibility to non-radiative centers. This could result in abovementioned higher PL efficiency for green wafer 1.

**Figure 6:**
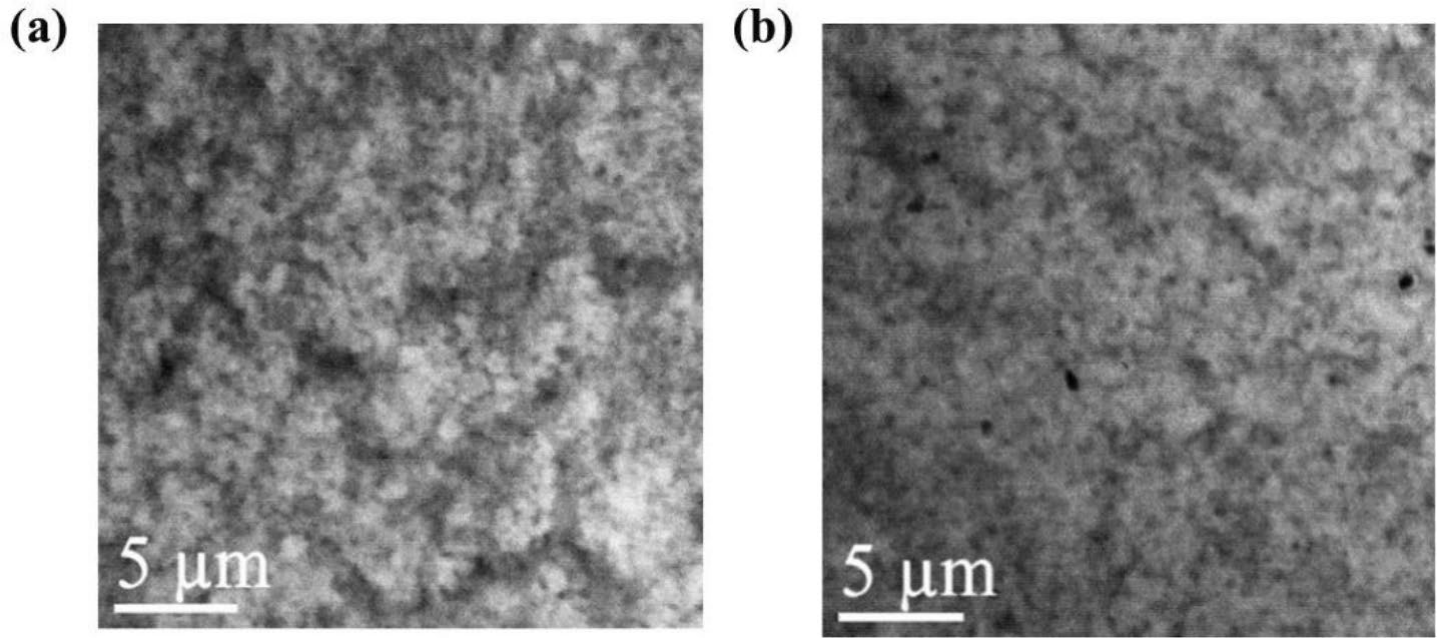
80 K monochromatic CL images taken at (**a**) 514 nm for green LED wafer 1 and (**b**) 522 nm for green LED wafer 2. Both wavelengths are center wavelengths for QWs of each wafer at 80 K.

To investigate the degree of indium localization in active region for the two green LED wafers, 80 K monochromatic CL images were taken at the center wavelength of each wafer, as shown in Fig. 6. A few micrometer sized indium segregation can be observed from green wafer 1, while it is relatively smaller (or even negligible) for green wafer 2. Combining the higher thermal activation energy and the higher degree of indium segregation, it is reasonable to conclude that green wafer 1 possesses larger and deeper local potential minima than green wafer 2. These could result in shorter non-radiative lifetime for green wafer 2, and smaller surface recombination velocity for green wafer 1 even both wafers underwent the same dry etching process.

## Conclusion

We investigated the efficiency change depending on the fabricated chip size in blue and green micro light-emitting diodes having different efficiencies in bare wafers. We suggested a key factor, ${v}_{s}/{\tau }_{0}^{-1}$, that determines the trend of size-dependent efficiency more precisely than the previously suggested surface recombination velocity alone. Although two green LED wafers are mainly compared with a blue LED wafer in this work, the formula relation derived would be valid regardless of center wavelength since all the information related to the active region including radiative and non-radiative recombination rates and surface recombination velocity is involved. Possible explanations on the reason why two green LED wafers show different properties are given based on temperature-dependent PL and CL image analysis. With the suggested factor, more accurate prediction to the chip-size dependent efficiency of an LED wafer can be provided, which allows us to wisely evaluate proper wafers emitting various wavelengths for micro LED display applications.

## Methods

A blue LED wafer and two green LED wafers were grown on commercially available patterned sapphire substrates prepared by metal–organic chemical vapor deposition. Epitaxial structures of all three wafers were consisted of similar structures such as a n-GaN layer, InGaN/GaN superlattices, InGaN/GaN multiple QWs, and a p-type GaN layer. However, thickness of QWs and barriers and period of the QWs are differently optimized for blue and green LED wafers. For the blue LED wafer, 7 period of InGaN (3 nm)/GaN (7 nm) active region was grown, of which the first QW is n-type doped. For both green LED wafers, 6 period of InGaN (2.8 nm)/GaN (15 nm) active region is adapted, of which the first well is also n-type doped. Though two green LED wafers shared similar epitaxial structures, detailed growth conditions such as V/III ratio, growth temperature and pressure was changed, resulting in different wafer-level efficiencies. Various PL experiments were held in cryostat with closed-loop He cryogenic system. The temperature of the cryostat was controlled by PID temperature controller. A 405 nm pulsed laser with 2 MHz of repetition and an average power of 200 µW was chosen as the excitation source, prepared by pulse picking and frequency-doubling Ti:sapphire pulsed laser of which wavelength adjusted to 810 nm. A streak camera (Hamamatsu) was used to measure time-integrated and time-resolved PL signal. Micro LEDs with different chip sizes were fabricated by conventional photolithography followed by inductively coupled plasma reactive ion etching system. Cl_2_ and Ar gas with ratio of 3:1 were used for the etching with 250 W of RF power and 700 W of ICP power. The average etching depth of 1 µm was measured after the etching. For low temperature CL measurements, CL system equipped in scanning electron microscope (SEM) was cooled down to 80 K using liquid nitrogen.

## Data Availability

The data that support the findings of this study are available from the corresponding author upon reasonable request.
